# Zonotopic Linear Parameter Varying SLAM Applied to Autonomous Vehicles

**DOI:** 10.3390/s22103672

**Published:** 2022-05-11

**Authors:** Marc Facerias, Vicenç Puig, Eugenio Alcala

**Affiliations:** 1 Autonomous Systems, Department of Electrical and Electronic Engineering, University of Manchester, Sackville Street Building, Manchester M1 3BB, UK; marc.faceriaspelegri@postgrad.manchester.ac.uk; 2 Institut de Robòtica i Informàtica Industrial (CSIC-UPC), Llorens i Artigas 4–6, 08028 Barcelona, Spain; eugenio.alcala@upc.edu

**Keywords:** autonomous driving, LPV modelling, optimal estimation, interval methods

## Abstract

This article presents an approach to address the problem of localisation within the autonomous driving framework. In particular, this work takes advantage of the properties of polytopic Linear Parameter Varying (LPV) systems and set-based methodologies applied to Kalman filters to precisely locate both a set of landmarks and the vehicle itself. Using these techniques, we present an alternative approach to localisation algorithms that relies on the use of zonotopes to provide a guaranteed estimation of the states of the vehicle and its surroundings, which does not depend on any assumption of the noise nature other than its limits. LPV theory is used to model the dynamics of the vehicle and implement both an LPV-model predictive controller and a Zonotopic Kalman filter that allow localisation and navigation of the robot. The control and estimation scheme is validated in simulation using the Robotic Operating System (ROS) framework, where its effectiveness is demonstrated.

## 1. Introduction

In the last few years, there has been a strong development in the automotive area towards making cars autonomous. A vast number of lines of research can be found, covering the perception of the environment to control strategies that drive the car through a given environment. In this work, the autonomous driving problem is narrowed, focusing on the localisation of the vehicle. From the control perspective, one of the most interesting techniques in autonomous driving is Model Predictive Control (MPC). We aim to use this technique and complement it with an appropriate localisation algorithm; thus, the work from Alcalá et al. [1], a state-of-the-art LPV-MPC, was used as the control technique. This decision was motivated by the promising results obtained in the past with the mentioned control technique, which are enhanced through the application of LPV models, allowing solving the optimisation problem in a linear manner.

The purpose of this paper is to enhance the reliability of localisation algorithms by means of introducing interval calculus in the estimation, motivating the usage of Zonotopic Kalman Filters (ZKF), as presented in Combastel [2], which were extended to the LPV framework in this work. Similar techniques have already been explored; for instance, Yu et al. [3] used ellipsoids instead of zonotopes, proving an enhanced performance with respect to classical Kalman filters. It can be seen that the main advantage of this line of work is that by dealing with both noise and disturbances in an interval manner, there is no need to make any assumption regarding their nature, which leads to systems that behave in a guaranteed manner as long as they are bounded, this being a more realistic restriction to meet in real applications. To our knowledge, this is the first study using LPV and dynamic models in a set-based manner within the autonomous driving localisation problem.

This paper presents a control estimation architecture for solving the autonomous driving problem in an unknown environment, taking advantage of optimal control theory. As the purpose of this research does not include the development of an exploration algorithm, the proposed scheme is integrated with a spline-based path planning module, presented in Alcalá et al. [1] and tested in a simulated scenario using ROS.

## 2. Related Work

The localisation problem has been widely studied within the robotics field, and it is still an ongoing problem. However, it is worth noting that the main focus of the current research is on solving the feature detection, while applying well-known Kalman filters, Monte Carlo methods, or some of their variations, as presented in Singandhupe and La [4]. These techniques can be used with a wide variety of sensor arrays; for instance, Ramesh et al. [5] relied on using point cloud data to perform Simultaneous Localisation And Mapping (SLAM), while Bhamidipati and Gao [6] merged the information from both the camera and GPS, using zonotopes to enhance the robustness of the estimation. In another line of work, we can see that other research works aimed to isolate the localisation problem and used previously computed maps to simplify the problem. For instance, Wan et al. [7] used Kalman filters to fuse the data from GNSS, LiDAR, and an inboard sensor rig to locate the vehicle in a known environment.

This research was performed from a control systems point of view; thus, all the considerations related to sensing and computer vision are considered solved, even though they are under development. As presented in Cadena et al. [8], most of the localisation techniques implemented in the state-of-the-art approaches heavily rely on probabilistic Kalman filters or their variations, some of the most-popular implementations being the Extended Kalman filter (EKF), as e.g., the one proposed in Paz et al. [9], or FastSLAM, as for instance, the work of Roh et al. [10], which relied on a combination between Monte Carlo sampling methods and the EKF. Another research line approaches the estimation problem not from the probabilistic point of view, but based on interval calculus, some examples being Mustafa et al. [11] and Fabrice et al. [12]. Moreover, an interval version of the particle filter was proposed by [13], exploring how set theory can be used to enhance the robustness of the algorithm through applying the q-satisfied intersection. More recently, we have seen how zonotopes can be applied in manipulators to reduce the inherent uncertainty of probabilistic models, leading to more consistent estimates in Li et al. [14].

In terms of addressing the inherent linearisation problem in most of the SLAM algorithms, we can see that there have been attempts at solving it with similar techniques to the LPV approach proposed in this work; for instance, Guerra et al. [15] applied a similar approach to the nonlinear kinematic model of a tricycle robot; in another line of work, Pathiranage et al. [16] used fuzzy logic to address the nonlinearity of the sensor models. On the other hand, we can see how model switching can also be used to enhance the performance of the system when facing variable noise conditions, as can be seen in [17].

## 3. Background Material

In this section, the preliminary knowledge required for the formulation of the set-based state estimation scheme proposed in this paper is introduced. Firstly, the basic zonotope operations are presented, and secondly, a reduction method to address dimensionality issues is explored.

### 3.1. Zonotopes

Zonotopes are a class of convex polytopes defined as *p*-dimensional hypercubes in an $R^{n}$ space, formed by a centre, $c_{z}$, and a radius matrix, $R_{z}$:

(1) $$\left[Z\right]=c_{z}+R_{z}$$

In the following, the most relevant properties used can be found, as presented in paper [18]:

1. The sum of two zonotopes is denoted as the Minkowski sum, and for $z_{1}$ and $z_{2}$:
  (2) $$\left[Z_{3}\right]=\left[Z_{1}\right]+\left[Z_{2}\right]=c_{z_{3}}+R_{z_{3}}$$
  where
  $$\begin{array}{cccc} c_{z} & =c_{z_{1}}+c_{z_{2}} & \;R_{z_{3}} & =[R_{z_{1}}R_{z_{2}}] \end{array}$$
2. A unitary zonotope is defined by R=I with appropriate dimensions.
3. The convex hull of a zonotope is defined as the smallest centred interval vector containing $R^{nxp}$.
  (3) R=rs(R)
  where rs() denotes a row sum operator, which generates a diagonal matrix, whose elements are defined by:
  (4) $$rs{\left(R\right)}_{ii}=\sum_{j=1}^{p}\left|R_{ij}\right|$$

The motivation behind using these types of sets is that their basic operations can be easily handled as simple matrix manipulation, which ensures low computational costs when operating with them.

**Figure 1:**
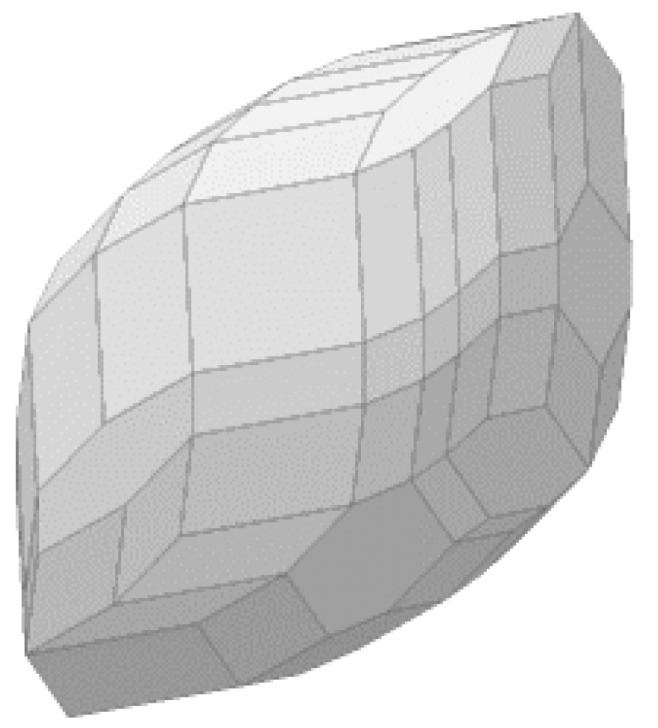
Graphical representation of a zonotope.

A visual representation of a zonotope can be found in Figure 1, obtained from [19]. It can be seen that a set in the space is approximated by a polytope, represented by using both $c_{z}$ and $R_{z}$, which are the central point of the polytope and its shape. In this way, we can apply the numerical tools presented in this section to propagate a given initial set under given conditions, being the robot inputs and the noise limits of the system. In this particular representation, we would expand the shape over time, generating a bounded set for each time instant *k* that encloses all the possible states of the system.

### 3.2. Dimensional Reduction of a Generation Matrix

As can be seen in the previous section, consecutive operations with zonotopes might lead to arbitrarily large matrices, which makes the implementation of a reduction strategy necessary. The algorithm used relies on a heuristic presented in [18] and is formalised in Algorithm 1.
**Algorithm 1** Dimensional reduction of a generation matrix.1: Define *d*, stating the maximal zonotope complexity2: Sort the column vectors *R* in decreasing order based on their euclidean norm, leading to:
$$R=[Q_{1}\cdots Q_{d-2}Q_{d-1}Q_{d}]$$3: Replace each set $\left[Q_{d-1}Q_{d}\right]$ by its interval hull.

## 4. Modelling

The vehicle used to implement the proposed algorithms is a 1:10 RC Car designed by the University of Berlin. It has been designed to allow an autonomous navigation and mapping by having a LiDAR, depth cameras, encoders, an IMU, and a GPS module. Furthermore, the vehicle can be actuated through a steering servo drive and a DC motor.

Due to the nature of the algorithms, a mathematical formulation of the behaviour of the system is required. In this section, the estimation model is presented. Firstly, the equations of the model are introduced, and secondly, those expressions are reformulated in an LPV manner. Note that both the vehicle and landmark behaviour are treated independently and ultimately are merged into a unique LPV model.

### 4.1. Nonlinear Vehicle Model

In this work, a dynamic bicycle model based on the approximation of a four-wheeled vehicle into a two-wheeled one is used, as proposed in [1]. This approach analyses the forces applied to the vehicle and derives a set of equations describing the dynamic behaviour of the system. The resulting equations are reformulated as a continuous time nonlinear model:

(5) $$\dot{x}=f\left(x,u\right)\;$$

where the state and control vectors, respectively, are defined as

(6) $$x=\left[\begin{array}{c} v_{x}\\ v_{y}\\ \omega \\ x\\ y\\ \theta \end{array}\right],\;u=\left[\begin{array}{c} \delta \\ a \end{array}\right]\;$$

Applying the bicycle model formulation leads to:

(7) $$\begin{array}{cc} \; & \dot{x}=v_{x}\cos\theta -v_{y}\sin\theta \\ \; & \dot{y}=v_{x}\sin\theta +v_{y}\cos\theta \\ \; & \dot{\theta }=\omega \\ \; & {\dot{v}}_{x}=a_{motor}-F_{df}+\frac{-F_{yf}\sin\delta }{m}+\omega v_{y}\\ \; & {\dot{v}}_{y}=\frac{F_{yf}\cos\delta +F_{yr}}{m}-\omega v_{x}\\ \; & \dot{\omega }=\frac{F_{yf}a\cos\delta -F_{yr}b}{I} \end{array}$$

where

(8) $$\begin{array}{cccc} \; & F_{yf}=C_{f}\alpha_{f} & \; & \alpha_{f}=\delta -atan\left(\frac{v_{y}+a\omega }{v_{x}}\right)\\ \; & F_{yr}=C_{r}\alpha_{r} & \; & \alpha_{r}=atan\left(\frac{b\omega -v_{y}}{v_{x}}\right) \end{array}$$

Additionally, a friction term $F_{df}$ is introduced to model the influence of the static friction force and drag force that act to oppose the movement of the vehicle. μ, ρ, and *g* are the static friction coefficient, the air density at 25 °C, and the gravity, respectively. $C_{d}$ is the product of the drag coefficient and vehicle cross-sectional area.

(9) $$F_{df}=\frac{\frac{1}{2}C_{d}\rho_{air}A_{f}{\left(v_{x}\right)}^{2}+\mu mg}{m}$$

State variables $v_{x}$, $v_{y}$, and ω represent the body frame velocities, i.e., linear in *x*, linear in *y*, and angular velocities, respectively. States *x*, *y*, and θ represent the world frame position coordinates as translations in both the *x* and *y* axis and a rotation with respect to the *z* axis. The control variables δ and *a* are the steering angle at the front wheels and the longitudinal acceleration vector on the rear wheels, respectively. $F_{yf}$ and $F_{yr}$ are the lateral forces produced in the front and rear tires, respectively. Front and rear slip angles are represented as $\alpha_{f}$ and $\alpha_{r}$, respectively, and $C_{f}$ and $C_{r}$ are the front and rear tire stiffness coefficients. *m* and *I* represent the vehicle mass and inertia, and $l_{f}$ and $l_{r}$ are the distances from the vehicle centre of mass to the front and rear wheel axes, respectively. All the dynamic vehicle parameters are properly defined in Table 1.

### 4.2. LPV Modelling of the Vehicle

An LPV model relies on redefining the expression presented in (5) by means of embedding the nonlinear nature of the equations into matrices that depend on a set of scheduling variables ϕ according to [20]:

(10) $$\begin{array}{cc} x_{k} & =A_{robot}\left(\phi \right)x_{k-1}+B_{robot}\left(\phi \right)u_{k-1}+w_{k}\\ y_{k} & =C_{robot}\left(\phi \right)x_{k}+v_{k} \end{array}$$

ϕ being a set of variables known as scheduling variables, which modify the value of the *A*, *B*, and *C* matrices to adapt them to the current vehicle operating point.

This technique allows expressing the system as linear with respect to both states and control actions by embedding the nonlinearities in the system matrices. In general, the system keeps being nonlinear; however, by instantiating ϕ at a given time instant, it is possible to extend classical control techniques (as, e.g., LMIs) designed for linear systems:

(11a) $$A_{robot}\left(\phi \right)=\left[\begin{array}{cccccc} A_{11} & A_{12} & A_{13} & 0 & 0 & 0\\ 0 & A_{22} & A_{23} & 0 & 0 & 0\\ 0 & A_{32} & A_{33} & 0 & 0 & 0\\ \cos(\theta ) & -\sin(\theta ) & 0 & 0 & 0 & 0\\ \sin(\theta ) & \cos(\theta ) & 0 & 0 & 0 & 0\\ 0 & 0 & 1 & 0 & 0 & 0 \end{array}\right]\;,$$

(11b) $$B_{robot}\left(\phi \right)=\left[\begin{array}{cc} -\frac{1}{m}\sin\delta C_{f} & 1\\ \frac{1}{m}\cos\delta C_{f} & 0\\ \frac{1}{I}\cos\delta C_{f}a & 0\\ 0 & 0\\ 0 & 0\\ 0 & 0 \end{array}\right]\;,$$

being

(11c)A11=−μA12=Cfsinδmvx(11d)A13=Cfasinδmvx+vyA22=−Cr+Cfcosδmvx(11e)A23=−Cfacosδ−Crbmvx−vxA32=−Cfacosδ−bCrIvx(11f)A33=−Cfa2cosδ+b2CrIvx

where the vector of scheduling variables ϕ is defined by a combination of states and control inputs.

(12) $$\phi ={\left[\begin{array}{ccccc} v_{x} & v_{y} & cos(\theta ) & sin(\theta ) & \delta \end{array}\right]}^{T}$$

This formulation needs to be slightly modified to be used in further sections of this paper, as a discrete model is required. The discretisation procedure is trivial by approximating the derivative terms by their finite differences.

### 4.3. Landmark Modelling

In this section, the observation model of the system is derived considering that the vehicle provides us with the following measurement, *m*, which can be unequivocally related to the landmark *i*:

(13) $$m_{i}={\left[r_{i}\left(k\right),\alpha_{i}^{1}\left(k\right)\right]}^{T}$$

$r_{i}\left(k\right)$ being the distance between the centre of the vehicle and the landmark and $\alpha_{i}^{j}$ a bearing measurement. In order to simplify the definition of an observation model, these measurements are expressed as Cartesian coordinates related to the COG of the robot. This approach leads to

(14a) $$\begin{array}{c} x_{lm_{i}}^{r}\left(k\right)=-x_{r}cos\left(\theta \right)-y_{r}sin\left(\theta \right)+x_{lm_{i}}^{w}cos\left(\theta \right)+y_{lm}^{w}sin\left(\theta \right) \end{array}$$

(14b) $$\begin{array}{c} y_{lm_{i}}^{r}\left(k\right)=x_{r}sin\left(\theta \right)-y_{r}cos\left(\theta \right)-x_{lm_{i}}^{w}sin\left(\theta \right)+y_{lm}^{w}cos\left(\theta \right) \end{array}$$

In order to model the behaviour of the landmarks, we need to express the landmarks, $x_{lm_{i}}^{w}\left(k\right)$ and $y_{lm_{i}}^{w}\left(k\right)$, as part of a differential model, which, due to their static nature, have a zero derivative:

(15) $$\begin{array}{c} {\dot{x}}_{lm_{i}}^{w}=0 \end{array}$$

(16) $$\begin{array}{c} {\dot{y}}_{lm_{i}}^{w}=0 \end{array}$$

Finally, this formulation needs to be rewritten as an LPV model, the matrices $A_{lm_{i}}$, $B_{lm_{i}}$, and $C_{lm_{i}}$ being defined considering the set of states presented in Equation (17) and *N* being the number of landmarks considered by the model:

(17) $$x=[v_{x},v_{y},\omega ,x,y,\theta ,x_{lm_{1}}^{w},y_{lm_{1}}^{w},\ldots ,x_{lm_{N}}^{w},y_{lm_{N}]}^{w}$$

(18) $$\begin{array}{cc} \; & A_{lm_{i}}=\left[\begin{array}{ccccc} 0 & 0 & \ldots & 0 & 0\\ 0 & 0 & \ldots & 0 & 0 \end{array}\right] \end{array}$$

(19) $$B_{lm_{i}}=\left[\begin{array}{c} 0\\ \vdots \\ 0 \end{array}\right]$$

(20) $$C_{lm_{i}}=\left[\begin{array}{cccccccc} 0 & 0 & 0 & -cos(\theta ) & -sin(\theta ) & 0 & cos(\theta ) & sin(\theta )\\ 0 & 0 & 0 & sin(\theta ) & -cos(\theta ) & 0 & -sin(\theta ) & cos(\theta ) \end{array}\right]$$

### 4.4. LPV Modelling of the System

It is straightforward to generate a differential model, as presented in Equation (10) by considering Equations (11) and (16). The resulting model can be reformulated in an LPV manner:

(21a) $$A\left(\phi \right)=\left[\begin{array}{cccc} A_{robot} & 0 & \ldots & 0\\ 0 & A_{lm_{1}} & \ldots & 0\\ \vdots & \vdots & \vdots & \vdots \\ 0 & 0 & \ldots & A_{lm_{N}} \end{array}\right]$$

(21b) $$B\left(\phi \right)=\left[\begin{array}{c} B_{robot}\\ 0\\ \vdots \\ 0 \end{array}\right]$$

(21c) $$C\left(\phi \right)=\left[\begin{array}{ccccc} C_{robot} & 0 & 0 & \ldots & 0\\ 0_{1,6} & C_{lm_{1}} & 0 & \ldots & 0\\ \vdots & \vdots & \vdots & \ldots & \vdots \\ 0_{1,6} & 0 & 0 & \ldots & C_{lm_{N}} \end{array}\right]$$

ϕ being a new set of scheduling variables,

(22) $$\phi ={\left[\begin{array}{ccccc} v_{x} & v_{y} & cos(\theta ) & sin(\theta ) & \delta \end{array}\right]}^{T}$$

Both terms $w_{k}$ and $v_{k}$ are related to the disturbances and sensors, respectively, and their covariances are defined by *Q* and *R*.

(23a) $$Q=\left[\begin{array}{cccc} Q_{robot} & 0 & \ldots & 0\\ 0 & 0 & \ldots & 0\\ \vdots & \vdots & \vdots & \vdots \\ 0 & 0 & \ldots & 0 \end{array}\right]$$

(23b) $$R=\left[\begin{array}{cccc} R_{robot} & 0 & \ldots & 0\\ 0 & R_{y_{1}} & \ldots & 0\\ \vdots & \vdots & \vdots & \vdots \\ 0 & 0 & \ldots & R_{y_{N}} \end{array}\right]$$

where $Q_{robot}$ and $R_{robot}$ are the noise covariance matrices associated with the vehicle.

Finally, the state vector is formed by the six original states of the model and two extra states for each landmark, while the output vector has the five original states and two additional ones for each landmark:

(24a) $$\begin{array}{c} x=[v_{x},v_{y},\omega ,x,y,\theta ,x_{lm_{1}}^{w},y_{lm_{1}}^{w}\ldots ,x_{lm_{N}}^{w},y_{lm_{N}}^{w}] \end{array}$$

(24b) $$\begin{array}{c} y=[v_{x},\omega ,x,y,\theta ,x_{lm_{1}}^{r},y_{lm_{1}}^{r},\ldots ,x_{lm_{N}}^{r},y_{lm_{N}}^{r}] \end{array}$$

This formulation is dimensionally varying, as in an unknown environment, the number of observer landmarks is not fixed, which makes it unsuitable with LMI design techniques. A solution to this issue is fixing the number of landmark processes each time to one and then replacing the information depending on which landmark is observed. This assumption holds as long as we consider the vehicle position independent of the landmark measurements.

## 5. Localisation Algorithm

In this section, the formulation of a zonotopic observer of an uncertain discrete dynamic system is presented, including the algorithm used and its implementation in a real system.

### 5.1. Algorithm

The aim of using zonotopic observers is the possibility of expressing the prediction as a region in an $R^{n_{x}}$ space defined by a zonotope. This region embeds all the possible states reachable by the robot given certain bounds on both measurement and system noise. In this approach, the model can be written as follows:

(25a) $$\begin{array}{c} x_{k}=Ax_{k-1}+Bu_{k-1}+E_{w}w_{k}, \end{array}$$

(25b) $$\begin{array}{c} y_{k}=Cx_{k}+E_{v}v_{k} \end{array}$$

As this is a set-based approach, both noise sources are defined as bounded by a unitary hypercube centred at the origin:

(26a) $$\begin{array}{c} w=\langle 0,I_{n_{w}}\rangle , \end{array}$$

(26b) $$\begin{array}{c} v=\langle 0,I_{n_{v}}\rangle \end{array}$$

Similarly, the set of states is represented using zonotopes.

(27) $$X=\langle c_{x}^{io},R_{x}^{io}\rangle$$

The equations of this observer can be derived by defining a Kalman filter where the Gaussian pdfs are replaced by zonotopic sets.

(28a) $$\begin{array}{c} {\hat{x}}_{k+1}=A{\hat{x}}_{k}+Bu_{k}+E_{w}w_{k}+L\left(y_{k}-{\hat{y}}_{k}\right) \end{array}$$

(28b) $$\begin{array}{c} {\hat{y}}_{k}=C{\hat{x}}_{k}+E_{v}v_{k} \end{array}$$

Then, by substituting Equation (28a) in Equation (28b), the expression of $\hat{x}$ can be easily generated:

(29) $${\hat{x}}_{k+1}=\left(A-LC\right){\hat{x}}_{k}+Bu_{k}+E_{w}w_{k}+-LE_{v}v_{k}+Ly_{k}$$

Finally, by applying the properties of zonotopic operators, the expressions of both the centre and generator matrix are defined as follows:

(30a) $$\begin{array}{c} c_{x}^{io}=c_{p}^{io}+L\left(y_{k-1}-Cc_{p}^{io}\right), \end{array}$$

(30b) $$\begin{array}{c} R_{x}^{io}=\left[\left(I-LC\right){R_{p}}^{io↓}-LE_{v}\right] \end{array}$$

where:

(31) $$\begin{array}{c} c_{p}^{io}=Ac_{x}^{io}+Bu_{k-1},R_{p}^{io}=\left[A{R_{x}}^{io↓}E_{w}\right] \end{array}$$

In this set of equations, the operator ↓ is used to symbolise a dimensional reduction of the zonotope. The presented structure requires the computation of an observer gain, which can be obtained by minimizing the $F_{W}$ radius of ${\langle c_{x}^{io}}_{p}$, ${R_{x}^{io}}_{p}\rangle$. This was covered in depth in [2].

### 5.2. Design Technique

The gain of the observer is computed by exploiting the LPV formulation of the system. One of the advantages of this type of formulation is that it allows designing the optimal Kalman gain at each of the vertices defined by extreme values of the scheduling variables (see Table 2).

Defining those limits leads to the computation of $2^{5}$ steady-state Kalman gains, which are derived by means of the LMI in Equation (32), presented in [21]. It is remarkable that the purpose of this computation is to derive optimal estimation gains for the estimator and, furthermore, ensure the stability of the algorithm by means of embedding stability conditions inside within the following LMI:

(32a) $$\begin{array}{c} \left[\begin{array}{cccc} -Y & YA_{i}-W_{i}^{T}C_{i} & YQ^{T} & W_{i}\\ A_{i}^{T}Y-C^{T}W & -Y & 0 & 0\\ QY & 0 & -I & 0\\ W^{T} & 0 & 0 & -R^{-1} \end{array}\right]<0, \end{array}$$

(32b) $$\begin{array}{c} \left[\begin{array}{cc} \gamma I & I\\ I & Y \end{array}\right]>0 \end{array}$$

The solution of this LMI is obtained by finding *Y* and $W_{i}$ for each vertex *i*. Finally, each of those gains can be computed as $L_{i}={\left(W_{i}Y^{-1}\right)}^{T}$. Then, a Kalman gain can be derived at each operational point by applying a weighted interpolation:

(33a) $$\begin{array}{cc} \mu_{i}\left(\phi \right) & =\prod_{j=1}^{N}\xi \left(\alpha_{j},\beta_{j}\right) \end{array}$$

(33b) $$\begin{array}{cc} \alpha_{j} & =\frac{\bar{\phi_{j}}-\phi_{j}\left(k\right)}{\bar{\phi_{j}}-\underline{\phi_{j}}} \end{array}$$

(33c) $$\begin{array}{cc} \beta_{j} & =1-\alpha_{j} \end{array}$$

ξ being the function computing all possible combinations and *N* the number of scheduling variables in ϕ. This allows the definition of *L* as

(34) $$\begin{array}{c} L\left(\phi \right)=\sum_{i=1}^{2^{N}}\mu_{i}\left(\phi \right)L_{i} \end{array}$$

### 5.3. Designing towards a Practical Implementation

It is clear that due to the nature of the system, there are two subsystems clearly differentiated, which represent both kinematic and dynamic behaviours. During preliminary experiments, it was found that the rate at which this state evolves is dramatically different, and in order to maintain a proper estimation of the kinematic states, the algorithm had to run at a frequency of around 200 Hz. This could lead to performance problems in certain robots; thus, it was decided to explore a cascade architecture, which allows considering both the dynamics and kinematics of the vehicle independently.

In order to do so, firstly, the state vector defined in Equation (24) needs to be split in two parts, the dynamic system being defined by

(35a) $$\begin{array}{c} x_{d}=\left[v_{x},v_{y},\omega \right] \end{array}$$

(35b) $$\begin{array}{c} y_{d}=\left[v_{x},\omega \right] \end{array}$$

and the kinematic dynamic system being defined by

(36a) $$\begin{array}{c} x_{k}=\left[x,y,\theta ,x_{lm_{1}}^{w},y_{lm_{1}}^{w},\ldots ,x_{lm_{N}}^{w},y_{lm_{N}}^{w}\right] \end{array}$$

(36b) $$\begin{array}{c} y_{k}=\left[x,y,\theta ,x_{lm_{1}}^{r},y_{lm_{1}}^{r},\ldots ,x_{lm_{N}}^{r},y_{lm_{N}}^{r}\right] \end{array}$$

This implies a redefinition of the matrices *A*, *B*, and *C* for each subsystem considering as the inputs of the dynamic system the outputs of the kinematic one. Those matrices are redefined as follows:

(37) $$\begin{array}{ccc} A_{d}=A[1:3,1:3] & B_{d}=B[1:3,1:2] & C_{d}=C[1:3,1:3] \end{array}$$

(38) $$\begin{array}{cccc} A_{k} & =A[3:m,3:m] & C_{k} & =C[1:3,1:3] \end{array}$$

(39) $$\begin{array}{cc} B_{k} & =\left[\begin{array}{ccc} cos(\theta ) & -sin(\theta ) & 0\\ sin(\theta ) & cos(\theta ) & 0\\ 0 & 0 & 1\\ 0 & 0 & 0\\ 0 & 0 & 0\\ -1 & 0 & 0\\ 0 & -1 & 0 \end{array}\right] \end{array}$$

*m* being the dimension of the *A* matrix.

Once both subsystems have been uncoupled, it is trivial to apply the design methodology presented in Section 5.2. Then, they are implemented in a cascade manner, taking into account the need to have a temporal correspondence between both of them, ensuring that the estimation of the dynamic states is aligned with the measurements of the kinematic ones.

## 6. Implementation

**Figure 2:**
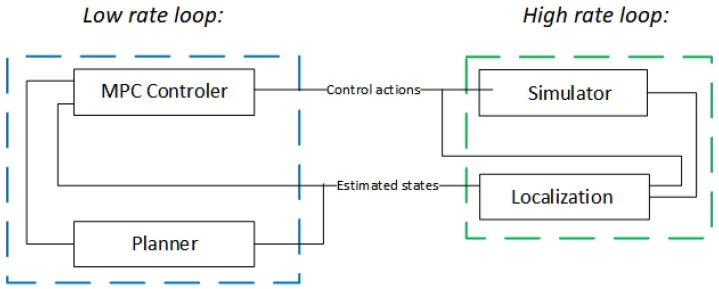
Proposed solution outline.

In this section, the implementation of the proposed navigation algorithm within the autonomous driving framework is proposed. Due to the nature of the problem that we wanted to address, the whole system was implemented in ROS. This ensured a proper simulation and the scalability of the results into a real platform, as it is trivial to port the implementation of the codes into the physical RC Car. Furthermore, it provides a realistic temporal behaviour of the detection hardware. A scheme of the proposed platform is represented in Figure 2, which represents the layout of the experiments performed in this work.

It can be seen that the system is divided into three main parts: control, simulation, and localisation. Each of these parts is described in the following.

### 6.1. Experimentation Environment and Simulation

**Figure 3:**
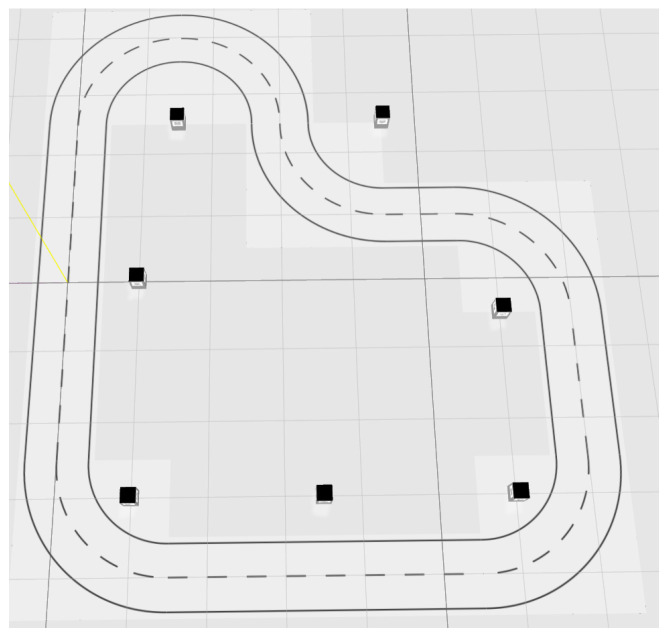
Simulation environment (small dark squares represent the landmarks).

The simulation platform relies on a numeric model of an RC Car, which simulates the dynamic behaviour of the robot, allowing the navigation through the scenario presented in Figure 3, with a representation of both the path to be followed and the landmark. In the typical SLAM problem, where an exploration algorithm is used to map the unknown environment, it is decided to use a known track and then locate the landmarks along the path.

The resulting system relies on a numerical simulation of the dynamical system using the model presented in Section 4 with noise added to both the control actions and the model states. This computation updates the position of the robot in the Gazebo environment. On top of that, we relied on gazebo to simulate landmark detection using a virtual camera and fiducial markers. The motivation behind this dual scheme is that the numeric model used to simulate the vehicle was extensively tested and tuned in [1]. Furthermore, we have perfect knowledge and control over the noise applied to the system, which is an important requirement due to the nature of the algorithms presented. The camera noise defined for this experiment was sampled from a uniform distribution bounded to ±0.1m. Noise involved in the system can be found in Equation (40), $E_{v}$ being associated with the measurements and $E_{w}$ associated with the model.

(40a) $$E_{v}=\left[\begin{array}{ccccc} 0.1 & 0.16 & 0.06 & 0.06 & 0.17 \end{array}\right]$$

(40b) $$E_{w}=10^{-3}\left[\begin{array}{cccccc} 0.2 & 0.18 & 1.40 & 0.13 & 0.16 & 0.068 \end{array}\right]$$

### 6.2. Control and Planning

The controller used to drive the car through the world presented in Section 6.1 is an MPC controller that uses an error-based dynamic model, as presented in [1], along with a spline-based planner, which exploits the fact that the car can be placed within the known track while detecting and estimating the position of each landmarks. This planning approach was covered deeply in [1].

### 6.3. Localisation

In this section, the implementation of the estimator presented in Section 5.1 is expanded into a localisation approach considering three different scenarios. On the one hand, we implemented an estimation that deals with the dynamic states of the system, which operates by solving Equations (29) and (30) with the subsystem associated with the velocity of the system.

On the other hand, we considered the kinematic estimation connected in a cascade framework, which deals with both robot and landmark position. Firstly, if no landmark is detected, the localisation algorithm behaves as a Kalman filter, using the information available from the on-board sensors, correcting the information provided by the system model without considering the terms that involve the landmark detection.

Secondly, if a non-registered landmark is detected, its position is set by considering the measurement as the real position of the system and instantiating it by applying Equation (14). Finally, if a registered landmark is detected, the model is updated accordingly, and then, the position of both the landmark and vehicle is updated by merging both the camera info and the rest of the on-board sensors. This strategy is stated in Algorithm 2.
**Algorithm 2** Landmark estimation algorithm.Initialisation**while** Robot is moving **do**    $data_{obs}\leftarrow Observation$    $data_{mov}\leftarrow Odometry$    **if** Landmark detected **then**        **while** Landmark list ≠empty **do**           **if** New landmark detected **then**               Add to the map the new location           **end if**           **if** Old landmark detected **then**               Load into the state vector the old location               Update system LPV matrices (22) )               LPV-KF $\leftarrow x_{k},u_{k}$               LPV-KF $\to x_{k+1}$               Store new estimation           **end if**        **end while**        **if** No landmark detected **then**           $x_{k}=x_{k}\left[1:6\right]$           LPV-KF $\leftarrow x_{k},u_{k}$           LPV-KF $\to x_{k+1}$        **end if**    **end if**    Update robot position, and wait until next movement**end while**

## 7. Experiments and Results

This section is devoted to the assessment of the proposed approach. The experiment consisted of two complete laps along the circuit proposed in Figure 3. The role of the control is to complete the laps tracking a certain cruising speed. On the other hand, the localisation algorithm provides an estimation of both the robot and landmarks detected along the path along with a region where the position of the robot is considered to be guaranteed.

During this experiment, both the control and estimation were decoupled in order to ensure that they did not interfere each other. It is worth noting that perfect data association was assumed; thus, we considered that the only source of uncertainties was the different noises present in each sensor and the non-perfect modelling, which are both defined as bounded without any prior knowledge of any distribution. Finally, a comparison between the proposed algorithm and a widely used localisation algorithm, the Extended Kalman Filter (EKF), is provided. It is worth noting that in order to keep a proper relation between both strategies, the EKF implementation was performed using LPV techniques in order to avoid Euler discretisation.

The EKF for this comparison was implemented with the same structure presented in Algorithm 2, the only difference being the state estimation, which is generated by applying Equation (41), where *A* and *C* are the model matrices, *Q* and *R* are the tuning parameters, and *L* is the gain matrix to be applied in the estimation. Predict:

(41a) $${\hat{x}}_{k}^{-}=A{\hat{x}}_{k-1}^{-}+Bu_{k-1}$$

(41b) $$P_{k}^{-}=AP_{k-1}A^{T}+Q$$

Update:

(41c) $$L_{k}=P_{k}^{-}C^{T}{\left(CP_{k}^{-}C^{T}+R\right)}^{-1}$$

(41d) $${\hat{x}}_{k}={\hat{x}}_{k}^{-}+L_{k}\left(y_{k}-C{\hat{x}}_{k}^{-}\right)$$

(41e) $$P_{k}=\left(I-L_{k}C\right)P_{k}^{-}$$

**Figure 4:**
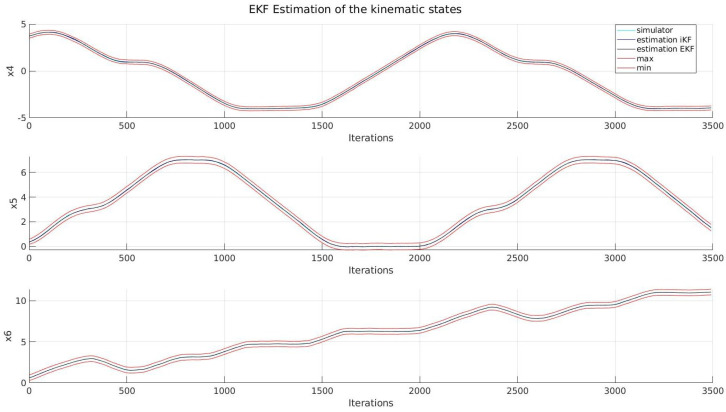
Kinematic states.

**Figure 5:**
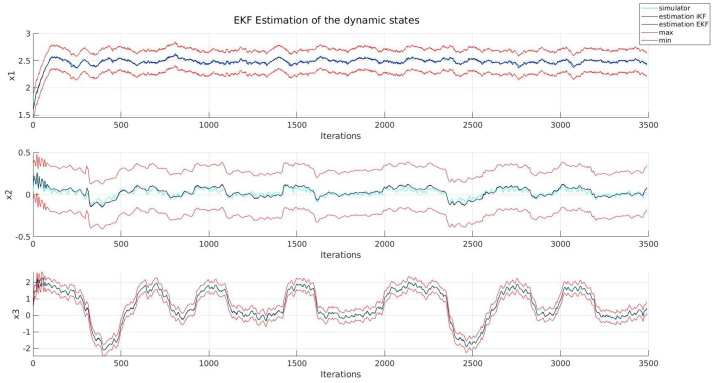
Dynamic states.

Firstly, in Figure 4 and Figure 5, the behaviour of the kinematic and dynamic variables of the system can be seen, the performance of both implementations being very similar, as in terms of the RMSE, the EKF and its set-membership version both present similar values, as can be seen in Table 3. This phenomenon was expected, as according to Combastel, both estimators are equivalent as long as certain conditions are met. However, as the noise distributions are not assumed when applying intervals, the resulting region will bound the state of the system under any circumstance, which does not apply to an LPV EKF.

**Figure 6:**
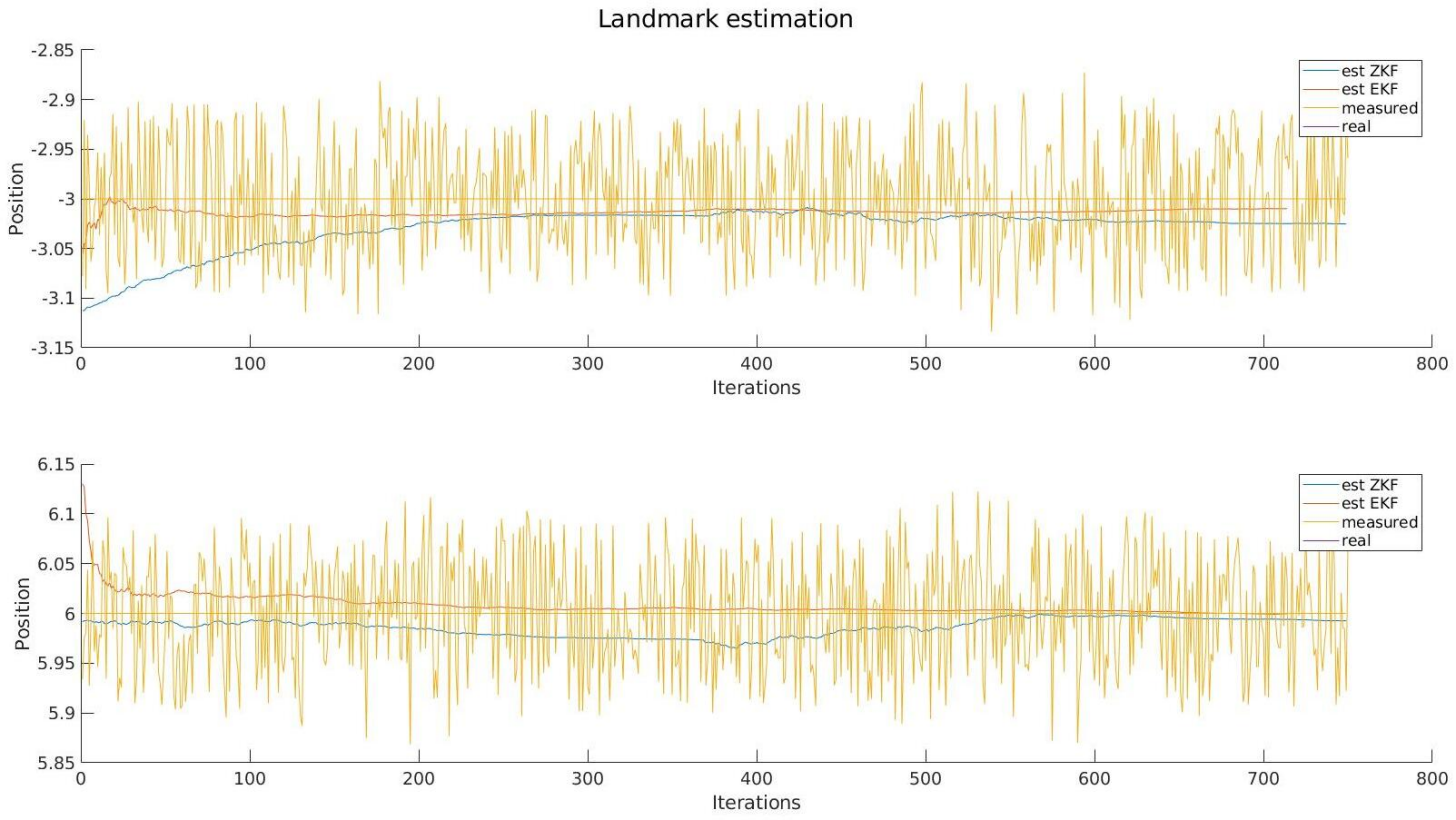
Landmark estimation.

**Figure 7:**
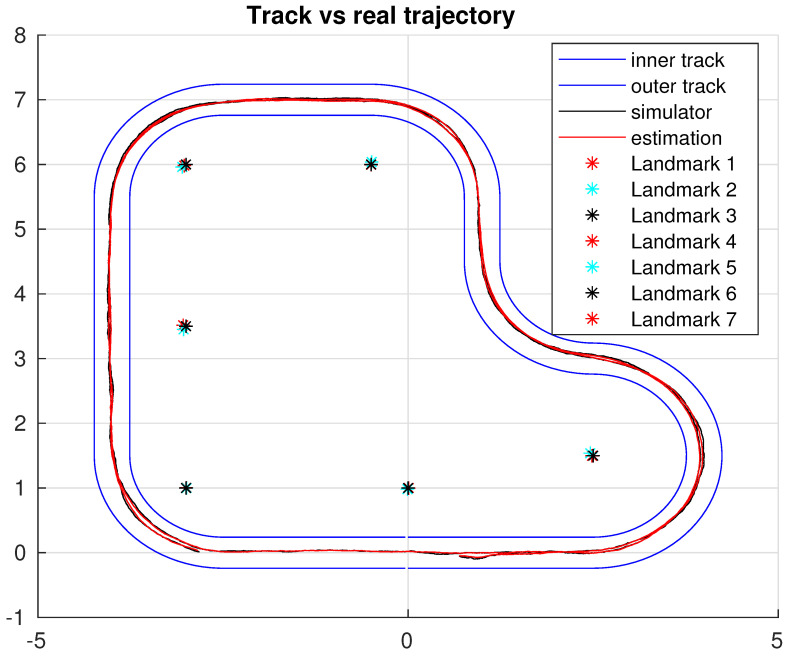
Path along the system.

Secondly, we can see the resulting estimation of all the landmarks detected during the path; it can be seen that the discussion presented before holds for the rest of the system, and in terms of accuracy, it presents the same behaviour. It is worth noting that due to the similarities between each figure, only one landmark was included, which can be found in Figure 6, while the general behaviour can be seen in Figure 7.

When comparing both implementations, it can be said that the most remarkable difference between both approaches is that the region that restricts the position does not depend on any assumed property of the noise other than its bounds, overcoming in this way one of the limitations of probabilistic implementations of the EKF.

In addition, tests in different conditions to ensure the viability of the algorithm were performed. In particular, we tested how the noise conditions may degrade the performance by doubling the noise levels in the landmark location and diminishing the number of landmarks in the path traversed from seven to four. As can be seen in Table 4, where the results previously shown have been added as the baseline case, for all tested scenarios, the performance was similar, showing the robustness of the algorithm in different conditions.

## 8. Conclusions and Future Work

In this paper, a zonotopic LPV Kalman filter was proposed as an alternative to the classical EKF for SLAM applied to autonomous vehicles. The proposed approach was able to provide a robust estimation in scenarios where, by construction, probabilistic methods such as the EKF should find their performance trimmed while providing a certain bound to the states that can be used to enhance security in autonomous navigation. The results achieved motivate the usage of interval over probabilistic techniques within the framework studied, as being more flexible in terms of modelling, this ensures proper performance given any bounded noise.

The work presented in this paper opens the door towards enhancing the security of algorithms used within the autonomous driving field. As seen in the literature, most of the state-of-the-art techniques rely on assumptions and relaxations on the characterisation of both the vehicle and the noise, while we proposed a novel approach that is less constraining in this sense. On the one hand, applying LPV modelling allows having an exact linear representation of a nonlinear system. On the other hand, we were able to treat noise by only assuming known bounds. Furthermore, having guaranteed knowledge about the maximum and minimum state values at each time instant allows the design of navigation techniques that can traverse a given path while mathematically ensuring that no collisions will happen as long as the obstacle is not within the bounds of the estimation.

Along the development of this research, different lines of investigation out of the scope of the initial hypothesis appeared, and we consider the following to be the most interesting ones:

- Design control techniques that consider the intervals generated by the localisation to enhance the application safety.
- Create a framework able to adapt itself towards certain sensor failures by exploring localisation within the fault detection field.
- Explore how data-based algorithms could be used to improve the modelling of both the robot and noise.
- Evaluate the performance of the localisation algorithms under extreme circumstances.

In conclusion, we believe that the viability of enhancing probabilistic techniques by applying interval calculus, in particular zonotopes, was assessed and proven to be more flexible in terms of noise definition than other techniques in the field. In addition, the capability of both modelling and designing control estimation algorithms by means of applying LPV techniques is a feasible solution to deal with nonlinear systems within the autonomous driving field. 

## Figures and Tables

**Table 1:** Dynamic model parameters of the vehicle.

Parameter	Value	Parameter	Value
$l_{f}$	0.125 m	$l_{r}$	0.125 m
*m*	1.98 kg	*I*	0.03 kg m^2^
$C_{f}$	68	$C_{r}$	71
μ	0.05	ρ	1.225 kg m^3^
$C_{dA}$	0.03 m^2^	*g*	9.8 $\frac{m}{s^{2}}$

**Table 2:** Scheduling variables’ limits.

Scheduling Variable	Minimum	Maximum
$V_{x}$	0.1	3.5
$V_{y}$	−2	2
cos(θ)	−1	1
sin(θ)	−1	1
δ	−0.3	0.3

**Table 3:** Error comparison.

	$v_{x}$	$v_{y}$	*w*	*x*	*y*	θ
LPV EKF	0.0066	0.0747	0.0065	0.0019	0.0019	0.0010
ZKF	0.0088	0.0799	0.0066	0.0020	0.0021	0.0011

**Table 4:** Error in different scenarios.

	$v_{x}$	$v_{y}$	*w*	*x*	*y*	θ
Baseline case	0.0066	0.0747	0.0065	0.0019	0.0019	0.0010
Noise doubled	0.0071	0.1147	0.0074	0.0021	0.00264	0.0012
4 landmarks	0.0072	0.0676	0.0082	0.0041	0.0034	0.0016

## Data Availability

All the required data are included in the paper.
